# Association between physical fitness and perceived work ability among Finnish population: a cross-sectional study

**DOI:** 10.1007/s00420-024-02058-y

**Published:** 2024-03-25

**Authors:** Valtteri Pohjola, Katariina Sarttila, Markus Kuusela, Riku Nikander, Annamari Lundqvist, Jouni Lahti

**Affiliations:** 1 Finnish Institute for Health and Welfare, P.O. Box 30, 00271 Helsinki, Finland; 2 Faculty of Sport and Health Sciences, University of Jyväskylä, Jyväskylä, Finland; 3 Department of Physical Medicine and Rehabilitation, Central Hospital of Central Finland and Central Finland Wellbeing Services County, Jyväskylä, Finland

**Keywords:** Health-related fitness, Workability, Cross-sectional studies, Physical capacity, Occupational health

## Abstract

**Objective:**

This cross-sectional study aims to examine association between different components of physical fitness and perceived work ability among working age population.

**Methods:**

The population-based study sample included 2050 participants aged 18–74 from the Finnish national Health 2011 study. Physical fitness was assessed by the single leg stand test, the modified push-up test, the vertical jump test and the six-minute walk test, and perceived work ability was assessed via interview. Logistic regression was used for examining the associations between physical fitness and work ability.

**Results:**

After adjusting for potential confounders (age, sex, marital status, educational level, work characteristics, total physical activity, daily smoking, BMI and number of diseases), odds ratios indicated that good work ability was more likely among those who had better balance in single leg stand test (OR = 1.54; 95% CI 1.07–2.24), and who belonged in the high fitness thirds in six-minute walking test (OR = 2.08; 95% CI 1.24–3.49) and in vertical jump test (OR = 2.51; 95% CI 1.23–5.12) compared to lowest third. Moreover, moderate (OR = 1.76; 95% CI 1.02–3.05) to high fitness (OR = 2.87; 95% CI 1.40–5.92) in modified push-up test increased the likelihood of good work ability compared to lowest third.

**Conclusion:**

These study results indicate that good musculoskeletal as well as cardiorespiratory fitness are associated with better perceived work ability. Promoting physical fitness in individual and societal level may be potential targets for maintaining good work ability in working age population.

**Supplementary Information:**

The online version contains supplementary material available at 10.1007/s00420-024-02058-y.

## Introduction

Traditionally work ability has been defined as a balance between worker’s individual factors, such as health and physical functioning, and job demands (Ilmarinen 2009). Demands for these individual factors may differ whether work tasks require more mental or physical attributes. In recent decades the concept of work ability has also widened to more comprehensive models which emphasizes the importance of micro and macroenvironments, such as social network and health and occupational policies, outside the work in terms of individual work ability (Gould et al. 2008). Therefore, multidisciplinary studies are required to identify potentially modifiable factors promoting high quality working life.

Population ageing has led to an increase in official retirement age in several European Union countries as retirement age is linked to life expectancy. Moreover, the number of older workers has rapidly increased within the first two decades of twenty-first century and the increase is predicted to continue until 2050 (Eurostat 2020). Understandably the aging labour force has become a major concern for public health and labour policies due to the association between ageing and decrease in work ability (Gould et al. 2008). It has been suggested that this relationship between ageing and decrease in work ability could be due to physiological decline in aging worker’s performance, especially in physically demanding work tasks (Varianou-Mikellidou et al. 2020). In addition, poor work ability itself increase the risk of disability, longer absence periods from work (Bethge et al. 2021) and disability pensions (Jääskeläinen et al. 2016). Furthermore, the level of physical activity in Finnish adults is too low with respect to current Physical Activity Recommendations (Piercy et al. 2018) and recent longitudinal studies have established that decline in muscular fitness (MF) and cardiorespiratory fitness (CRF) appears in adults within all age groups (Lamoureux et al. 2019; Santtila et al. 2018). The level of physical activity among working age population has declined even further during COVID-19 pandemic partly due to increase in remote work prevalence (Koohsari et al. 2021). Musculoskeletal diseases are the most common work-related health problem regardless of occupation or sector (Kok et al. 2020), and musculoskeletal disorders associate with decreased work ability. Thus, the decreasing physical activity seems worrying in respect to musculoskeletal health and work ability.

The association between physical fitness (PF) and perceived work ability have been investigated in a few previous studies with somewhat conflicting results. While majority of the observational studies have shown a positive relationship between MF, CRF (Kaleta et al. 2004; Kolu et al. 2022; Lebde et al. 2020; Marzuca-Nassr et al. 2021), balance (Holland et al. 2015; Lebde et al. 2020) and work ability, some studies have not found significant associations (Sörensen et al. 2008), or associations were only observed among the elderly (Baldwin et al. 2017; Suorsa et al. 2022). Moreover, most studies have focused on certain occupational groups and utilized heterogenous PF tests with insufficient reliability. Due to lack of population-based samples, the generalizability of the previous results should be done with caution.

Public health care and occupational health care are in a key position to find out the possible modifiable factors that increase the risk for work ability deterioration in individual level. However, generalizable evidence about the factors of PF influencing work ability and validated and feasible PF tests are needed to detect these risk factors. The aim of this study is to investigate association between different components of PF and work ability among working age population.

## Methods

### Study design and participants

This study included Physical Activity and Fitness sub-sample of 2050 participants (18–74 years old) who participated in Health 2011, a population-based cross-sectional and longitudinal observational study conducted between 8/2011 and 12/2011 by Finnish National Institute for Health and Welfare. Health 2011 was a follow-up study for Health 2000 –study and main purpose of the study was to provide up-to-date information about health and functional capacity of adult populations (Lundqvist and Mäki-Opas 2016). In 2000, a two-stage stratified cluster sample of over 18 years old adults was drawn from the nationwide population register in Finland. In 2011, all members of the Health 2000 -study sample who were still alive, lived in Finland and had appropriate contact information received an invitation for Health 2011 study. Moreover, new random sample of 18–29 years old adults was included in Health 2011 to cover young adults. Health 2011 Physical Activity and Fitness sub-study sample consisted of 4898 individuals of which 51% (n = 2455) participated. The participants of the sub-sample completed a health screening questionnaire as part of their health examination prior the PF assessment. The participants only took part in those fitness assessments for which they did not have any contraindications (Lundqvist and Mäki-Opas 2016). Eventually, from the 2050 participants in the physical fitness tests, a valid fitness measurements ranged from 65% (modified push-up test) to 90% (single leg stand).

Health 2011 was approved by The Coordinating Ethics Committee of the Hospital District of Helsinki and Uusimaa (45/13/03/00/11). All participants signed informed consent before the participation.

### Work ability assessment

Information about the perceived work ability based on Work Ability Score (WAS) was gathered via interviews. Participants were asked to estimate their current work ability compared to lifetime best on a scale from 0 to 10, where a score of 0 represented full work disability and a score of 10 indicated work ability at its best (Lundqvist and Mäki-Opas 2016). WAS is widely used measure of perceived work ability and it has sufficient convergent validity with more detailed multi-item Work Ability Index (WAI) (El Fassi et al. 2013) and it is also suitable for individuals not in the labour force (Gould et al. 2015). Additionally, WAS has been shown to predict sickness absence from work (Ahlstrom et al. 2010) and disability pensions (Jääskeläinen et al. 2016). For descriptive analyses, the mean score, and the distribution (poor = 0–5, moderate 6–7, good = 8–9, excellent = 10) of WAS is presented according to covariates (Gould et al. 2008). Furthermore, in logistic regression analyses, WAS was dichotomized into decreased work ability (0–7) and good work ability (8–10).

### Physical fitness assessment

PF was assessed according to field-based Health Related Fitness Test Battery which includes tests for balance, MF and CRF. MF tests included measures for the maximal power of lower extremities and endurance of upper limb extensor muscles (Lundqvist and Mäki-Opas 2016) and CRF included the six-minute walk test (6MWT). For the statistical analyses, MF and CRF variables were divided into thirds according to tertile cut-points.

The balance was assessed with the single leg stand test that measures static balance utilizing reduction of lower extremity support area (Lundqvist and Mäki-Opas 2016; Suni et al. 1996). The test was performed with shoes on, and the participants were allowed to choose the preferred leg to stand on. The other leg was placed on the knee level against the medial side of the supporting leg and the knee was pointed outwards (hip externally rotated). Participants were asked to keep arms hanging relaxed on the side of participant’s body and keep their eyes open. Time was started when the participant reached the correct test position. The maximal duration of the balance test was 60 s and participants were able to perform the test twice unless the duration of the first trial was 60 s (Lundqvist and Mäki-Opas 2016). Since the majority (74%) of the participants reached the maximum duration, in the statistical analyses, the balance was dichotomized whether participants reached the maximum duration or not.

To assess the maximal power of lower extremities, the jump and reach test was performed (Lundqvist and Mäki-Opas 2016). The main purpose of jump and reach test is to measure the leg extensor muscle power (Suni et al. 1996). In the test, participants stood beside the jump-board facing towards the jump-board. At the beginning participants raised their magnesium powdered middle finger of the dominant arm against the jump-board to mark the standing height. After marking the standing height, participants were asked to jump vertically with maximal effort. Overall, one practice trial and two test trials were performed. The vertical difference between standing height and jumping height was measured in centimetres and the highest jump was the recorded score (Lundqvist and Mäki-Opas 2016).

For upper limb extensor muscle endurance assessment, the modified push-up test was conducted. The modified push-up test has been originally developed to evaluate the short-term endurance capacity of the upper extremity extensor muscles and individual’s ability to stabilize the trunk (Suni et al. 1996). In the test, participants started the performance lying prone on the mat. Push-up cycle began by participant clapping his/her hands behind the back once and after that they performed normal straight-leg push-up with elbows completely straight in the top-position and touching one hand to another. One push-up cycle ended back in the prone position. Each participant was allowed to practice different phases of push-up cycle once before a single test trial. The recorded test score was the total number of correctly performed push-ups in 40 s (Lundqvist and Mäki-Opas 2016). In addition, for participants who were unable to perform the modified push-up test, the dynamic sit-up test was used as a secondary measure of muscle strength (n = 406), but this subgroup was excluded from statistical analyses due to the low number.

CRF was assessed submaximally with the 6MWT (Lundqvist and Mäki-Opas 2016). The test was performed on 15 m long track using cones to mark the turning points at both ends of the track. Participants were instructed to walk the track back and forth for 6 min (running and jogging forbidden) as fast as they possibly could. The observer started timing when the participant began walking and counted the number of completed tracks during six minutes. Participant’s heart rate was monitored and asked after two and four minutes and checked at the end of the trial. The main measure, walking distance in meters, was calculated by multiplying the number of completed tracks plus the walked distance of the unfinished last round (Lundqvist and Mäki-Opas 2016).

### Covariates

In the Health 2011 -study, information concerning participant’s demographic factors such as age, sex and marital status were obtained from the Population Register Centre of Finland (Lundqvist and Mäki-Opas 2016). Other sociodemographic factors i.e., educational level and work characteristics were collected via interviews and self-administered questionnaires. Age was divided into three categories according to tertile cut-points (≤ 44yo, 45-58yo, ≥ 59yo) and marital status was classified as unmarried, married/in relationship, and divorced/widow. Educational level was classified into lower, intermediate, and high educational level based on the participant’s highest completed education. Work characteristics were divided into four categories (not employed, sedentary, light, physical) according to whether participants were in employment during the last 12 months and how much employed participants reported their work tasks include physically demanding activities or sedentary work.

Level of total physical activity (PA) was derived from the self-administered questionnaire that estimated average weekly physical activity during the last 12 months. Aerobic physical activity was determined as MET-hours/week including the amount of moderate to vigorous physical activity respondent participated in weekly basis. The amount of strength training was determined based on weekly strengthening and balance training sessions the respondent participated. For the statistical analyses, physical activity was further classified into four categories based on whether participant did not meet the current PA recommendations or met the current PA recommendations partly (met either the aerobic or strength recommendations but not both) or fully. Information concerning smoking was assessed by a single question concerning individual’s daily smoking habits. For statistical analyses, smoking was classified into three categories whether participant was non-smoker, occasional smoker, or daily smoker. Long-term illnesses were defined as a sum variable of all self-reported illnesses diagnosed by physician and divided into three categories whether participant had 0, 1–2 or 3 or more diagnosed diseases. Anthropometric measures such as weight, height, and body mass index (BMI (kg/m^2^)) were measured and calculated in the health examinations participants attended and BMI was classified into three categories (< 25 = normal, 25–29.99 = overweight, > 30 = obese).

### Statistical analyses

Data was analysed in IBM SPSS V29. To correct the distribution of known background factors among the participants to match the distribution of the study population, weighting was used. Weights were based on the inverse probability weighting method (Härkänen et al 2016; Lundqvist and Mäki-Opas 2016) and were calculated using register-based information for the entire sample concerning age, sex, marital status, education, geographical area, and native language. Normality of independent and dependent variables was assessed using Kolmogorov–Smirnov test and examining the distributions by numeric values and histograms.

Intergroup differences in WAS means were assessed using Mann–Whitney's U test for sex and single leg stand, and with Kruskall-Wallis' H test for the rest of the categorical variables. Background factors’ distributional differences within work ability categories was analysed using χ^2^ –test for all categorical variables. Logistic regression was used to examine PF indicators relationship with good work ability. In the multivariable analyses there were three adjusted models created: in the first model (Model^1^) the PF odds ratios were controlled for age and sex; in the second model (Model^2^) the odds ratios were controlled for age, sex and other sociodemographic factors (marital status, educational level, and work characteristics) and in the third model (Model^3^), health and lifestyle related factors (BMI, meeting PA recommendations, smoking and number of diseases) in addition to the aforementioned were included in the model. Collinearity between the included covariates was examined and the variance inflation factor values were below two, indicating no concern of multicollinearity in our models. For all statistical tests, p < 0.05 was considered a significant result.

Secondary analysis concerning the linear associations between PF variables and continuous WAS were assessed using Pearson’s correlation coefficient (Online resource 1) and general linear models (Online resource 2). Finally, to examine whether association between PF variables differ in relation to employment status or age group, we conducted two stratified analyses. At first, we performed the analysis (Model^3^) separately for participants who either reported having been in employment during the last 12 months (n = 1402) or did not (n = 606). Secondly, we conducted a stratified analysis (Model^3^) for all age groups (18-44yo, 45-58yo, 59 +). For all subgroup analyses, tertile cut-points for each PF variables were reclassified based on the subgroup distribution.

## Results

Participant characteristics in relation to background characteristics and PF are shown in Table 1 and Table 2. Mean WAS in the whole study population was 8.49 (SD 1.75). There were no differences in WAS between males and females. Of the study population 7.7% belonged to poor, 13.2% to moderate, 50.5% to good and 28.6% to excellent work ability group. Overall, the work ability was better in young adults versus older age groups, in normal BMI versus overweight and obese, within unmarried and married versus divorced or widowed, and among those with higher education compared to lower educational levels. In addition, the perceived work ability was better among employed versus unemployed, in physically non-strenuous work compared to physically strenuous work, in occasional smokers and non-smokers compared to daily smokers, and among those with fewer diagnosed diseases. Of the physical activity groups, those who met current PA recommendations had the highest WAS (mean 9.23) whereas those who partly met the recommendations did not differ from each other. Of the participants (n = 2050), valid PF test results were obtained from 87% in the 6MWT, 65% in the modified push-up test, 79% in the vertical jump test, and 90% in the single leg stand test. Results showed significant differences between work ability categories in relation to all PF attributes of the study population and the mean of WAS was consistently highest among the best PF thirds. Correlations between PF and WAS (Online resource 1) as well as estimated WAS means according to PF (Online resource 2) are shown in the supplementary material.

**Table 1:** Study population characteristics and factor distributions within work ability categories

Variables	Study population, N^c^ (%^d^)	WAS^a^, mean (SD)	Work ability category, N^c^ (%^d^)				
			Poor	Moderate	Good	Excellent	*p*
Total	2050	8.49 (1.75)	158 (7.7)	268 (13.2)	1029 (50.5)	582 (28.6)	
Age	2050						< 0.001
18–44	702 (48.4)	9.14 (1.31)	21 (2.2)	37 (5.3)	311 (41.9)	327 (50.6)	
45–58	675 (25.1)	8.45 (1.73)	42 (6.3)	60 (10.1)	369 (55.8)	201 (27.8)	
59 +	673 (26.5)	7.35 (1.85)	95 (15.1)	171 (25.8)	349 (51.5)	54 (7.6)	
		< 0.001					
Sex	2050						0.257
Female	1152 (51.7)	8.54 (1.75)	97 (6.9)	130 (10.4)	584 (47.5)	336 (35.3)	
Male	898 (48.3)	8.44 (1.74)	61 (6.4)	138 (13.6)	445 (48.5)	246 (31.5)	
		p = 0.125					
BMI^b^	2049						< 0.001
Normal weight	810 (43.6)	8.83 (1.48)	41 (4.0)	78 (8.7)	400 (45.5)	288 (41.8)	
Overweight	778 (36.0)	8.47 (1.65)	57 (6.0)	118 (14.1)	392 (48.7)	207 (31.2)	
Obese	461 (20.4)	7.77 (2.18)	60 (13.4)	72 (15.2)	236 (51.8)	87 (19.5)	
		< 0.001					
Marital status	2047						< 0.001
Unmarried	504 (38.7)	8.97 (1.43)	25 (3.5)	44 (7.6)	233 (42.0)	197 (46.9)	
Married/in relationship	1218 (48.6)	8.31 (1.83)	93 (8.0)	156 (12.6)	631 (51.7)	332 (27.7)	
Divorced /widow	325 (12.8)	7.71 (1.89)	40 (11.3)	66 (21.8)	164 (51.6)	53 (15.3)	
		< 0.001					
Education	1940						< 0.001
Low	143 (8.3)	7.08 (2.04)	24 (19.0)	48 (31.7)	60 (43.0)	10 (6.3)	
Intermediate	1274 (65.8)	8.12 (1.88)	124 (9.3)	183 (15.1)	659 (52.0)	307 (23.5)	
High	523 (26.0)	9.00 (1.17)	8 (1.0)	33 (6.2)	277 (51.4)	205 (41.4)	
		< 0.001					
Work characteristics	1996						< 0.001
Not employed	602 (30.2)	6.96 (2.17)	128 (21.3)	164 (27.2)	263 (43.7)	47 (7.8)	
Sedentary	631 (31.6)	9.07 (1.06)	8 (1.3)	32 (5.1)	323 (51.2)	268 (42.5)	
Light	315 (15.8)	9.02 (1.05)	5 (1.6)	18 (5.7)	168 (53.3)	124 (39.4)	
Physically strenuous	448 (22.4)	8.63 (1.47)	15 (3.3)	46 (10.3)	255 (25.3)	132 (29.5)	
		< 0.001					
Physical activity	1926						< 0.001
Not meeting recommendations	894 (43.1)	8.03 (1.91)	98 (10.4)	161 (17.5)	445 (48.7)	186 (23.4)	
Meets aerobic rec	547 (27.5)	8.53 (1.63)	36 (5.6)	50 (8.4)	286 (49.2)	172 (36.9)	
Meets strength rec	169 (9.2)	8.64 (1.68)	8 (4.8)	25 (11.1)	87 (55.1)	138 (49.8)	
Meeting recommendations	316 (20.2)	9.23 (1.03)	7 (1.3)	21 (4.5)	148 (44.3)	138 (49.8)	
		< 0.001					
Smoking	2039						0.020
Daily	277 (14.5)	8.21 (1.81)	27 (7.2)	35 (16.4)	145 (53.4)	84 (23.0)	
Occasionally	123 (7.7)	8.90 (1.34)	7 (2.2)	18 (12.2)	54 (43.9)	73 (41.7)	
Non-smoker	1639 (77.8)	8.50 (1.76)	124 (7.0)	215 (11.1)	830 (47.4)	800 (34.6)	
		< 0.001					
Number of diseases	2039						< 0.001
0	622 (38.9)	9.30 (0.88)	3 (0.2)	28 (3.6)	289 (43.3)	301 (52.9)	
1–2	924 (41.9)	8.41 (1.64)	52 (5.6)	114 (14.2)	520 (53.6)	238 (26.6)	
3 +	493 (19.2)	7.01 (2.22)	103 (22.1)	126 (23.8)	220 (45.2)	43 (8.8)	
		< 0.001					

^a^Work Ability Score

^b^Body Mass Index (kg/m^2^)

^c^Presented as unweighted counts of observations

^d^Proportions presented based on weighted counts

**Table 2:** Study population characteristics according to physical fitness

Physical fitness variables	Study population, N^c^ (%^d^)	WAS^a^, mean (SD)	Work ability category, N^c^ (%^d^)				
			Poor	Moderate	Good	Excellent	*p*
6MWT^b^ (m)	1781						< 0.001
Low	595 (29.8)	7.60 (2.02)	84 (14.8)	120 (20.2)	295 (50.1)	92 (14.9)	
Moderate	598 (33.0)	8.77 (1.33)	17 (2.7)	63 (10.7)	333 (51.1)	180 (35.5)	
High	588 (37.2)	9.19 (1.12)	8 (1.0)	30 (4.6)	289 (44.5)	259 (49.9)	
		< 0.001					
Modified push-up	1342						< 0.001
Low	459 (30.9)	8.40 (1.54)	24 (4.8)	63 (14.9)	267 (56.0)	103 (24.3)	
Moderate	532 (36.8)	8.95 (1.28)	12 (2.2)	47 (8.0)	276 (47.3)	193 (42.5)	
High	351 (32.4)	9.32 (0.89)	0	17 (4.0)	158 (42.3)	173 (53.7)	
		< 0.001					
Vertical jump	1626						< 0.001
Low	549 (27.4)	7.99 (1.86)	55 (11.1)	79 (14.9)	301 (54.0)	111 (20.1)	
Moderate	553 (34.7)	8.84 (1.31)	15 (2.4)	58 (11.0)	285 (48.0)	191 (38.6)	
High	524 (37.9)	9.14 (1.06)	7 (0.9)	34 (5.3)	266 (47.8)	214 (46.0)	
		< 0.001					
Single leg stand	1851						< 0.001
< 60 s	664 (31.2)	7.72 (1.97)	79 (13.5)	136 (20.1)	340 (50.2)	105 (16.2)	
60 s	1187 (68.8)	8.88 (1.43)	40 (3.0)	95 (7.7)	609 (48.0)	435 (41.3)	
		< 0.001					

^a^Work Ability Score

^b^Six-minute walk test

^c^Presented as unweighted counts of observations

^d^Proportions presented based on weighted counts

The univariable and adjusted odds ratios are shown in Table 3. In the logistic regression analyses, the analytical sample varied between 58–89% of the original sample size. Compared with the lowest fitness third in the age and sex adjusted model (Model^1^), better performance in all PF tests significantly increased the odds of reporting a good work ability. Odds ratios ranged from 1.89 (95% CI 1.24–2.89) in intermediate third in the modified push-up test to 6.25 (95% CI 3.46–11.31) in the highest third in the vertical jump test. In the second model (adjusted for age, sex, marital status, educational level, and work characteristics), better fitness compared with the lowest fitness third increased the odds of reporting good work ability in the 6MWT, the modified push-up test, and the single leg stand, OR’s ranging from 1.79 (95% CI 1.18–2.72) in moderate fitness third in the 6MWT to 3.21 (95% CI 1.54–6.69) in the highest third in the modified push-up test. In addition, also belonging in the highest fitness third in the vertical jump test (OR = 2.97; 95% CI 1.60–5.51) increased the likelihood for reporting good work ability compared with the lowest third. In the third model (adjusted for covariates in model 2 and smoking, PA, BMI and number of diagnosed diseases), better performance in the modified push-up test increased the odds of good work ability compared with the lowest third, OR’s ranging from 1.76 (95% CI 1.02–3.05) in the moderate fitness group to 2.87 (95% CI 1.40–5.92) in the high fitness third. In addition, also high fitness in the vertical jump (OR = 2.51; 95% CI 1.23–5.12) increased the likelihood of good work ability compared with the lowest third. Concerning CRF, belonging in the high fitness third in the 6MWT (OR = 2.08; 95% CI 1.24–3.49) increased the odds of good work ability compared with the lowest third. Finally, those who reached maximum duration in the single leg stand test had greater odds of reporting good work ability compared with those who did not (OR = 1.54; 95% CI 1.07–2.24).

**Table 3:** Factors of physical fitness associated with work ability. Presented as univariable and adjusted odds ratios

		OR_crude_		Model^1^		Model^2^		Model^3^
Physical fitness variable	N^b^	OR (95% CI)	N^b^	OR (95% CI)	N^b^	OR (95% CI)	N^b^	OR (95% CI)
6MWT^a^ (m)	1763		1763		1637		1605	
Low		1.00 (ref.)		1.00 (ref.)		1.00 (ref.)		1.00 (ref.)
Moderate		3.48 (2.30–5.26)		2.44 (1.60–3.72)		1.79 (1.18–2.72)		1.53 (0.97–2.41)
High		9.07 (5.64–14.60)		5.61 (3.48–9.06)		2.93 (1.80–4.79)		2.08 (1.24–3.49)
Modified push-up test	1328		1328		1214		1191	
Low		1.00 (ref.)		1.00 (ref.)		1.00 (ref.)		1.00 (ref.)
Moderate		2.16 (1.47–3.17)		1.89 (1.24–2.89)		1.87 (1.11–3.17)		1.76 (1.02–3.05)
High		5.67 (2.94–10.97)		4.93 (2.46–9.87)		3.21 (1.54–6.69)		2.87 (1.40–5.92)
Vertical jump	1612		1612		1483		1453	
Low		1.00 (ref.)		1.00 (ref.)		1.00 (ref.)		1.00 (ref.)
Moderate		2.22 (1.45–3.41)		2.06 (1.29–3.29)		1.55 (0.98–2.45)		1.39 (0.84–2.32)
High		5.19 (3.33–8.09)		6.25 (3.46–11.31)		2.97 (1.60–5.51)		2.51 (1.23–5.12)
Single leg stand (60 s)	1830		1830		1697		1662	
No		1.00 (ref.)		1.00 (ref.)		1.00 (ref.)		1.00 (ref.)
Yes		4.31 (3.13–5.94)		2.13 (1.57–2.89)		1.81 (1.31–2.49)		1.54 (1.07–2.24)

^1^Controlled for age and sex

^2^Controlled for age, sex, marital status, work characteristics and educational level

^3^Controlled for age, sex, marital status, work characteristics, educational level, recommendations related to PA, BMI, smoking and number of diseases

^a^Six-minute walk test

^b^Presented as unweighted counts

### Sensitivity analysis

The results of the employment status (Online Resource 3) and age group (Online Resource 4) stratified analyses can be found in supplementary materials. In the first sensitivity analysis, results remained primarily the same. Within employed population, better fitness regarding CRF and MF increased the odds of good work ability with slightly higher estimates compared with the full population results. However, the association between balance and work ability was not statistically significant. In the not employed population, better CRF, vertical jump and balance were associated with good work ability whereas association concerning modified push-up test and work ability was not significant. Age group stratified results indicated that better CRF increased the odds of good work ability in middle-aged and older age groups whereas moderate fitness in modified push-up test compared to lowest third increased the likelihood of good work ability within the young adults. Moreover, balance abilities associated with better work ability only in middle-aged adults.

## Discussion

Results of the present study showed that perceived work ability was better among individuals with higher PF. Furthermore, higher PF in terms of each MF, CRF and balance increased independently of sociodemographic, lifestyle and health-related factors, the likelihood of having good work ability among working age population.

In previous studies concerning the association between PF and perceived work ability, focus has mainly been on certain occupational (Ezzatvar et al. 2021; Sörensen et al. 2008) or age groups (Kaleta et al. 2004; Kolu et al. 2022; Padula et al. 2013; Smolander et al. 2010; Sörensen et al. 2007) and the range of used PF tests is broad, which weakens the generalizability of the results. This study strengthens the findings of previous literature by using a large random sample of Finnish population and widely used health-related fitness tests.

Our finding that better CRF in indirect submaximal test associated with higher perceived work ability is consistent with previous literature (Kolu et al. 2022; Lebde et al. 2020; Sörensen et al. 2008; Suorsa et al. 2022) as the independent association was found in full study population and in both employment status and age group stratified analyses. Submaximal exercise tests without cardiopulmonary fitness testing can provide sufficient estimates of CRF in a field-based setting (Mänttäri et al. 2018; Ross et al. 2016) and be feasible in population-based large sample studies (Ross et al. 2016). In longitudinal studies, there are limited indications that better CRF might prevent the deterioration of work ability (Pohjonen 2001) and decrease the risk for sickness absence periods (Strijk et al. 2011). Previous literature contains some inconsistency, but recently there has been some convincing findings in population-based settings concerning the association between CRF and perceived work ability. Baldwin et al. (2017) and Lebde et al. (2020) showed a moderate correlations between the CRF and Work Ability Index (WAI) in the middle-aged and older adults among Australian population. In addition, Husu et al. (2023) showed recently that CRF (6MWT based VO_2_peak) was associated with better perceived work ability among over 40 years old Finnish working population which is congruent with our study results according to subgroup analyses. Considering that poor CRF has become more common worldwide (Lamoureux et al. 2018) and CRF’s important role in individual health (Ross et al. 2016), work ability (Kolu et al. 2022; Lebde et al. 2020) and indirect health-care costs (Kolu et al. 2022; Wang et al. 2021) it will be a major future challenge for social- health- and labour policy.

Our findings concerning the positive relationship between muscular fitness (MF) and balance and perceived work ability are in line with previous literature (Lebde et al. 2020; Suorsa et al. 2022; Padula et al. 2013). Most of the previous studies have found the similar low to moderate correlation between MF variables and perceived work ability. Furthermore, there is limited evidence that a good MF and balance abilities may prevent the work ability deterioration in 5-year follow-up in home care workers (Pohjonen 2001). Additionally, in several previous studies better balance abilities have correlated with better work ability (Baldwin et al. 2017; Lebde et al. 2020; Marzuca-Nassr et al. 2021) but this association has occurred mainly among older adults and the significance of this linear relationship tends to decrease when controlling the association for other possible explanatory variables. In previous literature, the MF has been independently associated with better work ability among older adults (Suorsa et al. 2022) and this could be described somewhat expected as lower work ability has been hypothesized to partly result due to physiological decline related to aging process that might affect the ability to cope with especially physical work tasks (Ilmarinen 2001). However, in our subgroup analyses based on age groups, we found that the association between MF and work ability is not only ageing-related phenomenon since the association was found in the youngest age group as well.

According to the present study and previous literature, there is a growing need for effective ways to improve the PF to maintain the good work ability among aging labour force. At this point, only a small amount of RCT studies have investigated the effects of PF promotion on work ability and the results are conflicting. Overall, most of the studies have found that interventions focused on PF promotion improves the PF itself (Gram et al. 2012; Stenner et al. 2020), but there is uncertainty whether these improvements have positive effect on perceived work ability. In the future studies, the potential causal relationships between our observed association need to be further examined in longitudinal settings. Furthermore, more evidence is needed on effective ways to promote working aged population’s general health and work ability through lifestyle-related factors such as physical activity, weight management and PF.

## Methodological considerations

Strengths of the present study are large random sample that is representative sample of Finnish working aged adults. Sample consisted of participants with heterogeneous demographic backgrounds and non-participation was handled by weighting of observations to restore the representativeness of the data. Moreover, the comprehensive data based on population-level random sample made it possible to control the relationship with wide range of other explanatory variables. In general, the PF tests used in this study, such as vertical jump test and 6MWT are reliable and feasible to implement in epidemiologic research. Finally, WAS has been shown to be valid measure of work ability by predicting sickness absence from work (Ahlstrom et al. 2010) and disability pensions (Jääskeläinen et al. 2016).

There are some limitations in this study. At first, due to the cross-sectional design causal inferences cannot be drawn from these results. Secondly, while our study aimed to analyse a representative sample, it is important to acknowledge the non-participation rate and variability observed in the analytical sample proportions. The missing observations are mainly result of health screening to identify the subjects with contraindications for fitness testing and invalid measurements. Due to selection related to health screening, participants in this sub-sample were a bit healthier than Finnish population in general which likely led to more conservative associations. Consequently, it is advisable to consider the potential impact of the missing data on the generalizability of the results.

## Conclusion

In conclusion, we found that better musculoskeletal and cardiorespiratory fitness are associated with good work ability among working age population and recommend societal level actions for promoting healthy lifestyle such as physical activity to improve and maintain fitness and work ability of the population.

### Supplementary Information

Below is the link to the electronic supplementary material.

Supplementary file1 (DOCX 25 KB)

## Data Availability

The study data are available on reasonable request to the corresponding author and with permission from the Finnish National Institute for Health and Welfare (THL).
