# Supplementary material for: Association between physical fitness and perceived work ability among Finnish population: a cross-sectional study
Source: Int Arch Occup Environ Health. 2024 Mar 25;97(4):451–60. doi: 10.1007/s00420-024-02058-y (PMC10999380; doi:10.1007/s00420-024-02058-y)
Supplement: Supplementary file 1 — Supplementary file1 (DOCX 25 KB) [file 420_2024_2058_MOESM1_ESM.docx]

*Online Resource 1.* Physical fitness variables associated with work ability (WAS) in full study population, young (18-44), middle-aged (45-58 years) and older adults (59+ years). Association presented as Pearson’s correlation coefficients.

| Physical Fitness Variables | Study population | Young | Middle-aged | Older |
| --- | --- | --- | --- | --- |
| Cardiorespiratory fitness |  |  |  |  |
| 6MWT ^a^ (m) | 0.394** | 0.250** | 0.304** | 0.257** |
| Muscular fitness |  |  |  |  |
| Modified- push up test (repetitions) | 0.307** | 0.206** | 0.201** | 0.139* |
| Vertical jump (m) | 0.320** | 0.151** | 0.155** | 0.178** |
| Balance |  |  |  |  |
| Single leg stand | 0.360** | 0.108** | 0.165** | 0.195** |

* Significant correlation (p<0.05) with WAS

** Significant correlation (p<0.01) with WAS

^a^ Six-minute walk test

*Online Resource 2.* Estimated means of Work Ability Score with 95 % confidence intervals for physical fitness categories.

| PF variable | Model^1^ | | Model^2^ | | Model^3^ | |
| --- | --- | --- | --- | --- | --- | --- |
|  | N | Mean (95 % CI) | N | Mean (95 % CI) | N | Mean (95 % CI) |
| 6MWT ^a^ (m) | 1770 |  | 1644 |  | 1612 |  |
| Low |  | 7.77 (7.57-7.97) |  | 8.01 (7.81-8.21) |  | 7.96 (7.75-8.17) |
| Moderate |  | 8.59 (8.46-8.72) |  | 8.51 (8.33-8.68) |  | 8.35 (8.15-8.55) |
| High |  | 8.90 (8.76-9.04) |  | 8.68 (8.50-8.87) |  | 8.43 (8.19-8.67) |
| Modified push-up | 1333 |  | 1219 |  | 1196 |  |
| Low |  | 8.33 (8.18-8.47) |  | 8.29 (8.04-8.44) |  | 8.10 (7.88-8.33) |
| Moderate |  | 8.73 (8.59-8.86) |  | 8.57 (8.37-8.77) |  | 8.37 (8.15-8.58) |
| High |  | 9.07 (8.94-9.20) |  | 8.84 (8.61-9.06) |  | 8.53 (8.28-8.78) |
| Vertical jump | 1616 |  | 1487 |  | 1457 |  |
| Low |  | 7.94 (7.72-8.17) |  | 8.12 (7.91-8.34) |  | 8.02 (7.78-8.25) |
| Moderate |  | 8.57 (8.44-8.70) |  | 8.50 (8.32-8.68) |  | 8.30 (8.11-8.50) |
| High |  | 9.04 (8.89-9.20) |  | 8.79 (8.59-8.98) |  | 8.55 (8.33-8.77) |
| Single leg stand | 1839 |  | 1706 |  | 1671 |  |
| <60s |  | 8.02 (7.83-8.21) |  | 8.11 (7.88-8.34) |  | 8.07 (7.82-8.31) |
| 60s |  | 8.53 (8.42-8.64) |  | 8.45 (8.25-8.65) |  | 8.26 (8.02-8.50) |

^1^ Controlled for age and sex

^2^ Controlled for age, sex, marital status, work characteristics and educational level

^3^ Controlled for age, sex, marital status, work characteristics, educational level, recommendations related to PA, BMI, smoking and number of diseases.

^a^ Six-minute walk test

*Online Resource 3***.** Adjusted logistic regression analyses for cross-sectional associations between physical fitness and perceived work ability stratified by employment status.

| PF variable | Employed^1^ | | Not employed^1^ | |
| --- | --- | --- | --- | --- |
|  | N | OR (95 % CI) | N | OR (95 % CI) |
| 6MWT ^a^ (m) | 1000 |  | 481 |  |
| Low |  | 1.00 (ref.) |  | 1.00 (ref.) |
| Moderate |  | 2.50 (1.24-5.04) |  | 1.36 (0.78-2.37) |
| High |  | 1.54 (0.79-3.00) |  | 2.97 (1.53-5.76) |
| Modified push-up | 851 |  | 244 |  |
| Low |  | 1.00 (ref.) |  | 1,00 (ref.) |
| Moderate |  | 2.42 (1.11-5.27) |  | 1.57 (0.63-3.92) |
| High |  | 3.41 (1.36-8.51) |  | 1.39 (0.58-3.30) |
| Vertical jump | 957 |  | 384 |  |
| Low |  | 1.00 (ref.) |  | 1,00 (ref.) |
| Moderate |  | 1.52 (0.71-3.27) |  | 1.32 (0.61-2.87) |
| High |  | 4.78 (1.71-13.36) |  | 2.57 (1.04-6.33) |
| Single leg stand | 1036 |  | 497 |  |
| <60s |  | 1.00 (ref.) |  | 1.00 (ref.) |
| 60s |  | 0.91 (0.52-1.59) |  | 2.27 (1.27-4.08) |

^1^ Controlled for age, sex, marital status, work characteristics, educational level, recommendations related to PA, BMI, daily smoking and number of diseases.

^a^ Six-minute walk test

*Online Resource 4.* Adjusted logistic regression analyses for cross-sectional associations between physical fitness and perceived work ability stratified by age groups.

| PF variable | Young adults (<44)^1^ | | Middle-aged (44-58)^1^ | | Older adults (59+)^1^ | |
| --- | --- | --- | --- | --- | --- | --- |
|  | N | OR (95 % CI) | N | OR (95 % CI) | N | OR (95 % CI) |
| 6MWT ^a^ (m) | 500 |  | 554 |  | 551 |  |
| Low |  | 1.00 (ref.) |  | 1.00 (ref.) |  | 1.00 (ref.) |
| Moderate |  | 2.07 (0.70-6.13) |  | 1.49 (0.73-3.04) |  | 1.15 (0.67-1.99) |
| High |  | 0.64 (0.19-2.10) |  | 5.04 (1.80-14.12) |  | 2.32 (1.23-4.34) |
| Modified push-up | 455 |  | 445 |  | 291 |  |
| Low |  | 1.00 (ref.) |  | 1.00 (ref.) |  | 1.00 (ref.) |
| Moderate |  | 4.41 (1.46-13.33) |  | 1.11 (0.48-2.59) |  | 1.08 (0.50-2.30) |
| High |  | 2.53 (0.41-15.47) |  | 1.82 (0.57-5.77) |  | 1.12 (0.44-2.88) |
| Vertical jump | 487 |  | 530 |  | 436 |  |
| Low |  | 1.00 (ref.) |  | 1.00 (ref.) |  | 1.00 (ref.) |
| Moderate |  | 2.62 (0.73-9.38) |  | 0.96 (0.39-2.38) |  | 1.07 (0.55-2.07) |
| High |  | 5.53 (0.89-34.57) |  | 2.09 (0.62-7.08) |  | 1.83 (0.75-4.45) |
| Single leg stand | 513 |  | 575 |  | 574 |  |
| <60s |  | 1.00 (ref.) |  | 1.00 (ref.) |  | 1.00 (ref.) |
| 60s |  | 0.75 (0.19-2.89) |  | 1.92 (1.01-3.62) |  | 1.51 (0.90-2.54) |

^1^ Controlled for age, sex, marital status, work characteristics, educational level, recommendations related to PA, BMI, daily smoking and number of diseases.

^a^ Six-minute walk test
